# Expansion and Activation Kinetics of Immune Cells during Early Phase of GVHD in Mouse Model Based on Chemotherapy Conditioning

**DOI:** 10.1155/2010/142943

**Published:** 2010-12-21

**Authors:** Behnam Sadeghi, Suleiman Al-Hashmi, Zuzana Hassan, Bjorn Rozell, Hernan Concha, Carin Lundmark, Kjell-Olov Grönvik, Manuchehr Abedi-Valugerdi, Moustapha Hassan

**Affiliations:** ^1^Experimental Cancer Medicine, Institution for Laboratory Medicine, Karolinska Institutet, 141 86 Stockholm, Sweden; ^2^Center for Allogeneic Stem Cell Transplantation (CAST), Karolinska University Hospital, Huddinge, 141 86 Stockholm, Sweden; ^3^Morphology and Phenotype Analysis, Institution for Laboratory Medicine, Karolinska Institute, 141 86 Stockholm, Sweden; ^4^Department of Medicine, Center for Infectious Medicine (CIM) and Division of Hematology, Karolinska University Hospital, Huddinge, 141 86 Stockholm, Sweden; ^5^National Veterinary Institute, 75189 Uppsala, Sweden; ^6^Department of Biochemistry and Biophysics, Arrhenius Laboratories for the Natural Sciences, Stockholm University, 10691 Stockholm, Sweden; ^7^Clinical Research Centrum (KFC, Novum), Karolinska University Hospital, Huddinge, 141 86 Stockholm, Sweden

## Abstract

In the present paper, we have investigated early pathophysiological events in graft-versus-host disease (GVHD), a major complication to hematopoietic stem cell transplantation (HSCT). BLLB/c female mice conditioned with busulfan/cyclophosphamide (Bu-Cy) were transplanted with allogeneic male C57BL/6. Control group consisted of syngeneic transplanted Balb/c mice. In allogeneic settings, significant expansion and maturation of donor dendritic cells (DCs) were observed at day +3, while donor T-cells CD8+ were increased at day +5 (230%) compared to syngeneic HSCT. Highest levels of inflammatory cytokines IL-2, IFN-gamma, and TNF-alfa at day +5 matched T-cell activation. Concomitantly naïve T-cells gain effecr-memory phenotype and migrated from spleen to peripheral lymphoid organs. Thus, in the very early phase of GHVD following Bu-Cy conditioning donor, DCs play an important role in the activation of donor T cells. Subsequently, donor naïve T-cells gain effector-memory phenotype and initiate GVHD.

## 1. Introduction

Allogeneic hematopoietic stem cell transplantation (HSCT) is a curative therapy for the treatment of malignant and nonmalignant disorders. While graft versus leukemia (GVL) is promoted by donor T-cells [1] and desired when HSCT is used as a treatment for malignant disorders, the alloreactive donor T-cells that induce GVL effect may also initiate graft-versus-host disease (GVHD) [2–4]. GVHD is a serious complication that limits the use of allogeneic HSCT.

It has been reported that GVHD develops in three consecutive stages. (1) Inflammation coupled with a cytokine storm as result of pretransplant conditioning. (2) Activation of donor T-cells via recipient/donor antigen presenting cells (APCs). (3) Finally, damage of certain tissues by the activated donor T-cells [4–7]. Intestine, skin, liver, and lungs are the most frequently affected organs, which are assaulted by alloreactive donor T-cells [8].

Several investigations have shown that the occurrence and severity of GVHD depend on several factors, including the intensity of conditioning, the presence and number of donor T-cells in the graft, and the antigenic disparity between donor and recipient [9–12]. However, GVHD may occur in any type of allogeneic setting regardless of conditioning protocol [13, 14]. In both clinical settings [15] and in experimental models [3, 16], GVHD might occur long after DLI-induced GVHD or even without conditioning. These observations indicate the primacy and importance of immune competent cells in the pathophysiology of GVHD [3].

Understanding the cellular and molecular mechanisms underlying the initiation and development of acute GVHD is an important issue which can improve our knowledge and subsequently may help in providing strategies for the prevention and/or treatment of GVHD. Several studies have shown that certain recipient and/or donor cell populations [3, 17–19] and cytokines, for example, IFN*γ*, TNF*α* [20] are involved in the process of GVHD [4, 21]. However, to our knowledge, only few (if any) studies address the dynamics of donor and host immune cells expansion and activation pattern in combination with cytokine profile at the initiation stage of GVHD in a complete experimental setup. For instance, by utilizing an *in vivo* tracking system Beilhack et al. and Panoskaltsis-Mortari et al. have shown the migration pattern of donor cells in GVHD, but due to technical limitation they did not draw a dynamic model to include the interaction of different cell populations from donor and recipient source [6, 7].

Recently, we introduced a novel mouse model of GVHD based on chemotherapy conditioning [22]. In the present paper, we used this model to follow the dynamic of activation and proliferation pattern of donor immune cells in the secondary lymphoid organs of recipient during the early phase of GVHD. In parallel, the production of different proinflammatory cytokines was also evaluated.

## 2. Materials and Methods

### 2.1. Animals

Female BALB/c (H-2d) and male C57BL/6 (H-2b) mice, 10–12 weeks old were purchased from Scanbur (Sollentuna, Sweden). Mice were maintained under pathogen-free conditions with controlled humidity (55 ± 5%), 12 hours light/dark, temperature (21°C ±  2°C), and HEPA-filtered air. Animals were kept in individually ventilated cages and were fed autoclaved mouse chow and tap water *ad libitum.*

### 2.2. Bone Marrow Transplantation

Recipient mice underwent transplantation according to the protocol described previously [22]. Briefly, recipient female BALB/c mice received busulfan (80 mg/kg) for 4 days followed by cyclophosphamide (200 mg/kg) for 2 days. Day −1 and 0 considered as resting and BMT days, respectively.

Male C57BL/6 and female BALB/c mice were used as donors for allogeneic and syngeneic settings, respectively. At day 0, bone marrow cells (BMC) from donor femurs and tibias were flushed and single cell suspension was prepared. Spleen (SP) single cell suspension was prepared by disrupting the spleen. Cell number and viability was determined using the Trypan blue exclusion assay. Recipient mice were injected via the lateral tail vein with 2 × 10^7^ and 3 × 10^7^ cells of BMC and SP in a volume of 250 *μ*l. All experiments described here were approved by the South Stockholm ethics committee for animal research. Transplantation experiments have been repeated at three different time points.

### 2.3. Assessment of GVHD

Recipient mice were examined daily until the appropriate sampling day. Animals were evaluated for five clinical symptoms of GVHD: weight loss, posture, activity, fur texture, and skin integrity as described elsewhere [5, 22]. Liver, intestine, and skin were evaluated using histopathologic sections to confirm GVHD.

### 2.4. Cell Surface Staining for Flow Cytometry

Fluorescein isothiocyanate-(FITC) conjugated H-2K^b^(clone: AF6-88.5), H-2K^d^(clone: SF1-1.1), CD3 (clone: AF6-88.5), NK (clone: DX5), CD44 (clone: IM7), Ia-IE (clone: 2G9) and Phycoerythrin-conjugated (PE) conjugated H-2K^d^(clone: SF1-1.1), CD8 (clone: 53-6.7), H-2K^b^(clone: AF6-88.5) and PerCP-Cy5.5 conjugated CD3 (clone: 145-2C11), CD25 (clone: PC61), CD11b (clone: M1/70) and APC conjugated CD4 (clone: RM4-5), CD19 (clone: 1D3), CD62L (clone: MEL-14), and CD11c (clone: HL3) were purchased from Pharmingen (San Diego, California, USA). Spleen and bone marrow single cell suspension were prepared as described before [22].

For immunophenotyping, cells were first incubated with an FC-receptor blocking monoclonal antibody (clone: 2.4G2) for 15 min at 4°C and then directly stained with a panel of mAbs for 30 min at 4°C. Finally, the stained cells were washed twice with FACS buffer solution and analyzed with a FACS Calibur flowcytometer.

### 2.5. Cytokine Measurement

Blood (0.5–1 ml) was collected in an ependorf tube before killing the animals, serum was separated and kept at −80°C until analyzed using Gyrolab Bioaffy (Gyros AB, Uppsala, Sweden).

A Gyrolab Bioaffy CD contains 112 individual microstructures, each containing a 15 nl prepacked column with streptavidin-coated particles where the reaction takes place. Liquids (capture reagent, sample and detection reagent) are sequentially introduced into each microstructure using capillary force, either through an individual inlet or a common inlet that connects and serves eight microstructures via a common distribution channel. Each microstructure contains a volume definition chamber and an overflow channel enclosed by hydrophobic barriers. The volume definition chamber allows accurate metering within the CD of the sample portion intended for analysis (200 nl). Sample volume definition is incorporated as an integrated part of the analytical process avoiding problems such as evaporation and poor reproducibility commonly associated with metering of nanoliter volumes. The flow of liquid over the column is further controlled by centrifugal force created at appropriate spinning rates of the CD microlaboratory.

Gyrolab Workstation Control Software automatically controls the different steps when running a Gyrolab Bioaffy CD. Briefly, the individual streptavidin-coated columns are reconditioned by loading 0.01 M PBS, pH 7.2 containing 0.01% Tween 20 (PBS-T) through each common channel. The biotinylated capture antibody (500 nl at 100 *μ*l/ml) is then loaded through the common channel and 200 nl is spun over the capture bed for approximately1 min to saturate the streptavidin column. The column is then washed twice adding PBS-T through the common channel followed by rapid spinning. Protein calibrators to generate a reference curve or unknown samples are then added through the individual inlet holes into the microstructures (200 nl) followed by moderate spinning of the CD for approximately 3.5 minutes in order to slowly flow the 200 nl sample through the column to maximize capture of the cytokine. The column is rinsed twice by sequential addition of PBS-T followed by rapid spinning. An excess of detection reagent, a complementary antibody with a different epitope specificity and labeled with Alexa Fluor 647, is added through the common channel. The CD is spun again at a moderate rate to allow binding of the detection reagent to the captured cytokine. Finally the columns are washed 5 times with PBS-T and the CD is automatically transferred to the laser detection position where detection is carried out automatically using preselected detector settings for the laser-induced fluorescence (LIF, NeHe 633 nm) detector. Fluorescence data from each column in the CD is further analyzed with Gyrolab Evaluator software that is a Microsoft Excel add-in using XLFit (IDBS, Guildford, UK) for curve fitting. Gyrolab Evaluator software generates standard curves and calculates the concentrations of unknown samples. In addition, an image of the fluorescence intensity in each individual column can be displayed graphically by Gyrolab Viewer software to facilitate evaluation of assay performance and for investigation of any outliers.

The following capture antibodies were used: mab rat antimouse IL-2 clone JES6-1A12 (R and D System), mab rat anti-IFN*γ* clone R4-6A2 (BD Pharmingen), mab hamster anti-TNF*α* clone TN3-19.12 (RandD System), and for detection: mab rat antimouse IL-2 clone JES6-5H4 (RandD System), mab rat anti-IFN*γ* clone AN-18 (BD Pharmingen), and polyclonal goat anti-TNF*α* cat. nr AF-410-NA (R and D System). Recombinant cytokines produced in *E. coli* were used as standard proteins.

### 2.6. Histology and Immunohistochemistry

Tissue samples were fixed in neutral buffered formalin for 24 hr, transferred to 70% ethanol, dehydrated, and embedded in paraffin according to standard procedures. Sections of 4 *μ*m were prepared and stained using Hematoxylin and Eosin for histology evaluation.

For immunohistochemistry, tissues were embedded in OCT (Histolab, Stockholm, Sweden) and frozen in N-hexan cooled by dry ice. Immunohistochemical detection of CD4 (RM4-5), and CD8 (53-6.7) were performed using rat antimouse monoclonal antibodies (BD Pharmingen, San Diego, CA, USA). Briefly, 4-5 *μ*m sections were cut, fixed in cold (−20°C) acetone for 3 minutes, dried (overnight), rinsed with PBS, and treated with 3% H2O2 in methanol and blocked by 4% goat serum in PBS. Primary antibodies were diluted in the blocking solution and applied at 4°C for one hour. After rinsing in PBS, a biotin-labelled secondary goat antirat antibody was applied. Sections were incubated with ABC-HRP complex (BD Pharmingen, San Diego, CA, USA). Binding sites were visualized with diaminobenzidine/hydrogen peroxide, and the slides finally counterstained with hematoxylin.

### 2.7. Statistical Analysis

All data are expressed as mean ± S.E. (standard error) unless otherwise stated. Differences between allogeneic and syngeneic were analyzed using Mann-Whitney (*U*-test).

*P* < .05 is considered statistically significant. All statistical analyses were performed utilizing SPSS ver13.

## 3. Results

### 3.1. Myelo- and Lymphoablative Effects of Chemotherapy Conditioning on the Bone Marrow and Spleen

We investigated the effect of conditioning regimen (Bu-Cy) on the myeloid and lymphoid cells in the BM and spleen at day 0 (day of BMT). As shown in Table 1, treated mice exhibited a substantial decrease in the total numbers of bone marrow and spleen cellularity (95% and 63%, resp.). Moreover, except naïve and cytotoxic T-lymphocytes, all of the individual subpopulations in the bone marrow and spleen were reduced significantly in number by the treatment (Table 1). In BM, cells within both lymphoid (CD19+) and myeloid (CD11b+) lineages as well as dendritic (CD11c+) and natural killer (DX5, Pan-NK) cells were the most affected populations (Table 1) whereas in the spleen mostly NK, dendritic and B cells were decreased (Table 1). Nevertheless individual subpopulations were more sensitive to conditioning in the BM compared to spleen. In both organs, naïve T (CD44^low^CD62^high^)and cytotoxic T (CD8+) cells were the most resistant cells to the conditioning regimen (Table 1).

### 3.2. Recovery of the Bone Marrow and Spleen Cellularity at Early Phase of GVHD

**Figure 1:**
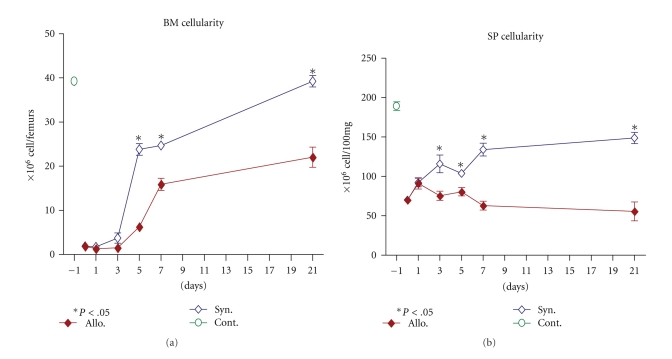
Bone marrow and spleen cellularity in allogeneic and syngeneic transplanted mice. Female BALB/c mice were transplanted with 20 × 10^6^ and 30 × 10^6^ BM and SP cells, respectively, from male C57BL/6 or BALB/c mice as allogeneic or syngeneic setting. Bone marrow (a) and spleen (b) cellularity at different time points after conditioning and transplantation. (a) Bone marrow cellularity calculated based on total cells extracted from both femurs. (b) Spleen cellularity determined based on 100 mg of spleen tissue. (*◆*): allogeneic group, (*◊*): syngeneic group and (O): control mice. All values are mean ± SE for 4 to 6 animals in each group per time point. Differences were analyzed statistically employing U-test, compared between groups (*P < .05).

Recently, we have shown that clinical and histopathological signs of GVHD started within 7 days after allogeneic BMT [22]. In the present study, we evaluated the recovery pattern in BM and repopulation of immune cells in spleen during the development and progress of GVHD, we followed the cellularity of bone marrow and spleen in allogenic and syngeneic grafts at different intervals (4–6 mice at each time point). In syngeneic recipient mice (Figure 1(a)), the recovery of BM cellularity was initiated at day +1, reached substantially high level on day +5 (>50% recovery), and was fully recovered by day +21. However, in allogeneic recipients, the bone marrow cellularity was delayed, had lower magnitude (Figure 1(a)), and did not recover until day +21 (Figure 1(a)). 

Similar to the BM, repopulation in the spleen of syngeneic recipient mice was rapid (started at day +1), increased by time (except for day +5, which showed a slight decrease), and reached to 75% of the control after 21 days (Figure 1(b)). In contrast, in allogeneic recipients, initial rapid increase of splenic cellularity at day +1 was observed, followed by severe decelerating until day +21. The numbers of splenocytes were about 25% of the control and 33% of the syngeneic recipients (Figure 1(b)).

### 3.3. Phenotype and Dynamics of the Recruited Immune Cells at the Beginning of GVHD

**Figure 2:**
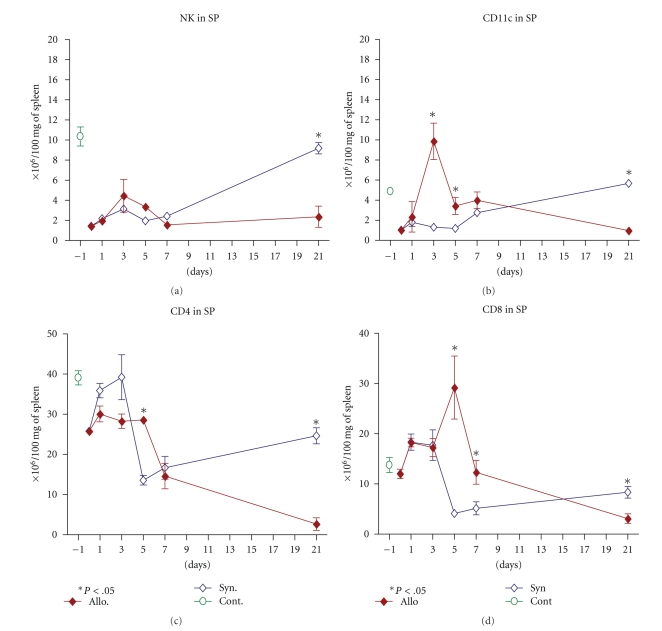
Immune cell phenotype and repopulation pattern in the spleen of allogeneic and syngeneic transplanted mice at the early phase of GVHD. Female BALB/c mice were transplanted with 20 × 10^6^ and 30 × 10^6^ BM and SP cells, respectively, from male C57BL/6 (allogeneic) or BALB/c (syngeneic) mice. Repopulations of different immune cells were evaluated in the spleen of recipient mice. Absolute number of (a) NK (DX5) (b) DCs (CD11c) (c) CD4 T helper and (d) CD8 cytotoxic T cells in 100 mg of the spleen tissue in allogeneic and syngeneic setting. (*◆*): allogeneic group, (*◊*): syngeneic group and (O): control mice. All values are mean ± SE for 4 to 6 animals in each group per time point. Differences were analyzed statistically employing *U*-test, compared between groups (*P < .05).

Several studies [6, 7] including ours [22] have shown that donor alloreactive cells proliferate in the secondary lymphoid organs at day +5 and invade target tissues at day +7. Thus, we characterized the phenotypes and activation status of the repopulated immune cells in the spleen of recipient mice shortly after BMT. As shown in Figure 2(a), in both allogeneic (GVHD) and syngeneic recipient mice, the absolute numbers of cells expressing DX5 (Pan-NK) slightly increased immediately after BMT (day +1) and expanded until day +3 showing more expansion in allogeneic compared to syngeneic group. These cells began to decline continuously in the allogeneic recipients and increased to reach the control level at day 21 posttransplantation in the syngeneic setting (Figure 2(a)).

Dendritic cells (DCs) have been shown to play an important role in triggering of GVHD [17, 18]. As shown in Figure 2(b), the absolute number of splenic DCs increased in both allo- and syngeneic setting one day after BMT. However, DCs were significantly (*P* < .01) higher (10-fold) in allogeneic transplanted mice compared to that seen in syngeneic and control groups (2-fold) (Figure 2(b)) at day +3. The higher number of DCs in the spleen of GVHD mice was persistent up to day +5 compared to that observed in the syngeneic group (*P* < .05). Seven days after BMT, the number of DCs in the spleen of allogeneic group started to decrease, while they recovered and reached to normal level by day +21 in syngeneic recipients (Figure 2(b)).

T-cell repopulation in the spleen of allogeneic and syngeneic transplanted mice showed that both groups exhibited an immediate and slight expansion of CD4+ and CD8+ T-cells up to three days after BMT (Figures 2(c) and 2(d)). The magnitude of CD4+ T-cell reconstitution was higher in syngeneic transplanted mice (*P* < .05). Nonetheless, at day +5, the population of CD4+ T-cells significantly decreased in the spleen of syngeneic group as compared to GVHD mice (*P* < .05).

In our previous investigation we have shown that cytotoxic CD8+ cells are the principal cell type that initiates GVHD and promotes tissue damage [22]. Interestingly, five days after BMT while T-cell subpopulations were decreasing in the spleen of syngeneic group, allogeneic transplanted mice exhibited a vigorous expansion of CD8+ T-cells (230% of control, *P* < .01). Thereafter, at day +7, the sizes of both populations (CD4+ and CD8+ T cells) declined and only reached to 10% and 30% of controls at day +21 whereas in syngeneic setting recovery of CD4+ and CD8+ T-cells reached close to normal level at day +21 (Figures 2(c) and 2(d)).

### 3.4. Donor DCs Repopulate and Maturate at Early Phase of GVHD

**Figure 3:**
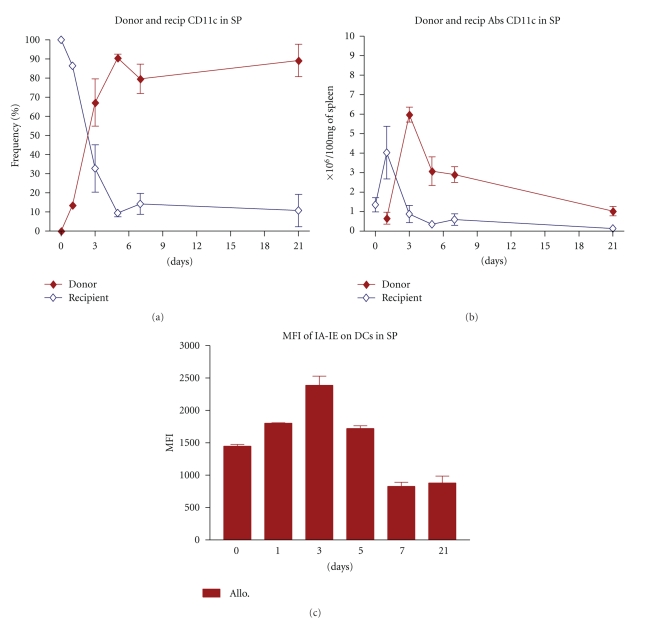
Dendritic cells chimerism and activation pattern in GVHD developing mice. Spleens of allogeneic transplanted recipients were evaluated for dendritic cell chimerism and maturation marker. (a, b) Frequency and expansion pattern of donor (CD11c+ and H-2Kb) versus host (CD11c+ and H-2Kd) dendritic cells in the spleen of recipient mice in GVHD developing setting. (c) Maturation status of DCs was evaluated using expression level of MHC class II on DCs surface. Three days after BMT donor DCs has higher expansion with more expression of maturation marker on cell surface. (*◆*): donor DCs (CD11c+ and H-2Kb), (*◊*): host DCs (CD11c+ and H-2Kd). All values are mean ± SE for 4 to 6 animals in each group per time point. Differences were analyzed statistically employing U-test, compared between groups (*P < .05).

The finding that the pattern of DCs repopulation in the spleen of allogeneic setting was strikingly different from that in the syngeneic transplanted mice (Figure 2(b)) raised two questions: firstly, are these cells of donor or recipient origin, and secondly, are these cells mature or activated? To answer these questions, we first analyzed the chimerism status in GVHD prone allogeneic recipients. As shown in Figure 3(a), while one day after the transplantation most of DCs (>85%) in the spleen have the recipient origin (CD11c+ H-2Kd+), at the time at which DCs expand intensively (day +3, Figure 2(b)), the majority (>65%) of these cells are donor derived (CD11c+ H-2Kb+) (Figures 3(a) and 3(b)).  Figure 3(b) represents the chronological pattern of host versus donor DCs expansion in the spleen of GVHD developing mice.

We further evaluated the activation (maturity) status of the identified DCs in the spleen by measuring the expression level of MHC-II (Ia-b) on these cells. As shown in Figure 3(c), the expression level of Ia-IEb (MHC-II) increased by time and reached the peak level (MFI = 2389) at day +3 after transplantation and thereafter reduced by time (Figure 3(c)).

### 3.5. Origin and Activation Status of Repopulated T Cells During the Initiation Phase of GVHD

**Figure 4:**
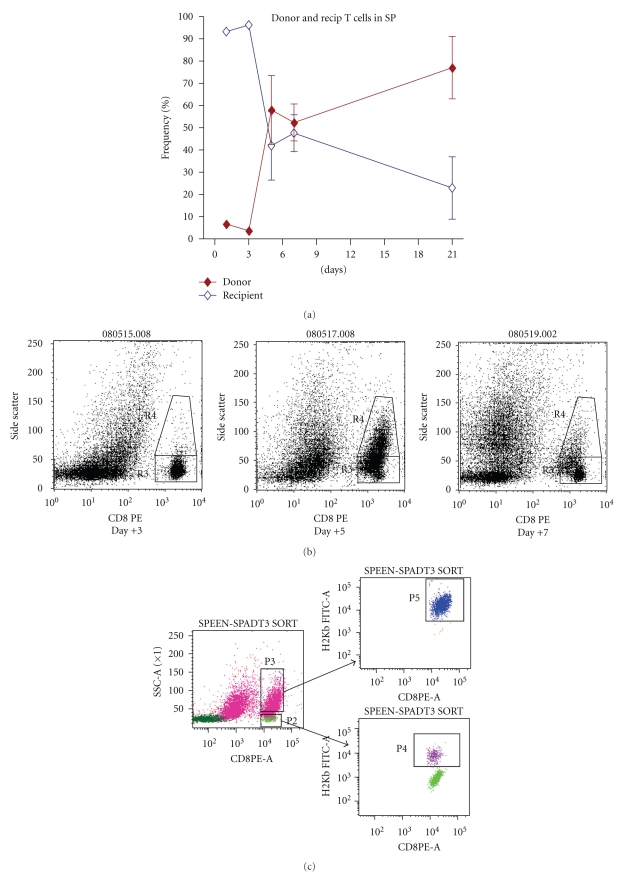

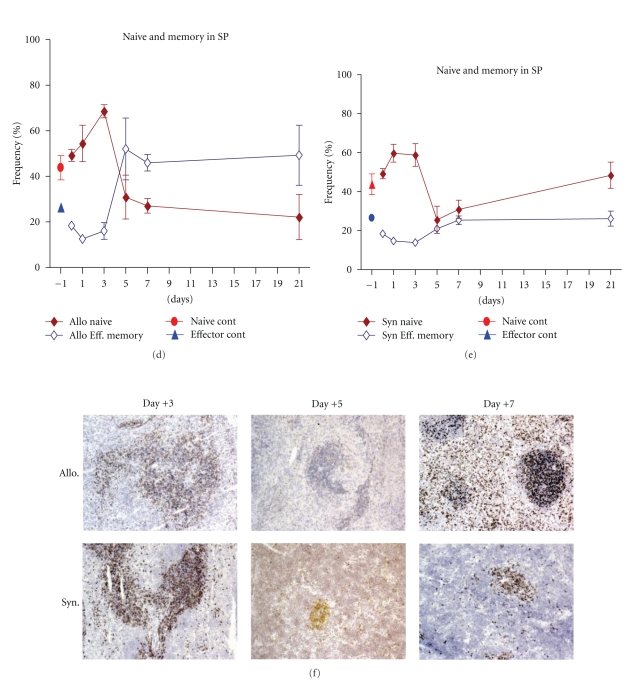
Chimerism and activation pattern of donor cytotoxic T cells in the spleen of GVHD mice. BALB/c mice were transplanted with 20 × 10^6^ and 30 × 10^6^ BM and SP cells from either allogeneic (C57BL/6) or syngeneic (BALB/c) donors. Spleens of transplanted recipients were evaluated using flowcytometry and histopathology methods. (a) Frequency of donor versus recipient T cells at different time points in the spleen of GVHD mice. (*◆*): donor (H-2Kb), (*◊*): host (H-2Kd) cells. (b) Large granular lymphocytes appeared 5 days after BMT in the spleen of GVHD mice however these cells decreased dramatically at day 7 after BMT. (c) Most of the granular large T cells (upper gate) at day +5 originated from donor. (d, e) Expansion pattern of naïve (CD44^low^CD62^high^) and effector memory (CD44^high^CD62^low^) T cells in the spleen of (d) allogeneic and (e) syngeneic transplanted mice. (f) Immunohistostaining of spleen in allogeneic (upper row) and syngeneic (lower row) setting indicate extensive spreading of CD8+ T cells (brown spots) in the spleen of allogeneic transplanted mice 5 days after BMT. (*◆*): naïve (CD44^low^CD62^high^), (*◊*): effector memory (CD44^high^CD62^low^) in allogeneic and syngeneic setting, (*⬤*): naïve (CD44^low^CD62^high^), (▲): effector memory (CD44^high^CD62^low^) in normal BALB/c mice. Differences were analyzed statistically employing *U*-test, compared between groups (*P < .05).

Dynamics of immune cell repopulation in the spleen of allogeneic transplanted mice showed that T-cells were increased by number (Figures 2(c) and 2(d)) at day +5 (two days after DCs maturation and expansion). Therefore, we evaluated this population to identify their origin and phenotype. As indicated in Figure 4(a) the frequency of donor T-cells increase from 3.7 ± 1.3% at day +3 to 58 ± 15.5% at day +5 after BMT. While donor T-cells were increasing during the transitional period (day +3 to +5), recipient T-cells decreased from 96.3 ± 1.3 to 42 ± 15.5 percent, respectively (Figure 4(a)).

We investigated the phenotype of T cells during GVHD development in the spleen of recipient mice. As shown in Figure 4(b), in allogeneic transplanted mice, a discernible population of CD8 T-lymphocyte emerges 5 days after transplantation. The new, granular, large lymphocytes appeared at day +5 and significantly decreased in number at day +7. Of interest, >95% of the large granular lymphocytes (upper gate) originate from the donor while small nongranulated lymphocytes (lower gate) were of mixed of donor and host origin (Figure 4(c)). Morphological analysis of the sorted donor CD8+ T-cells (CD8+, H-2b+) from both upper and lower gates showed that upper gate donor CD8+ cells have a larger nucleus and more cytoplasm comparing to lower gate cells (data not shown). Additionally ex vivo activation of these cells indicated that large granular lymphocytes proliferate more in response to Con-A stimulation (data not shown).

### 3.6. Donor Effector Memory T Cells Develop from Naïve T Cells during the Early Phase of GVHD

Both naïve (CD44^low^CD62^high^) and effector memory (CD44^high^CD62^low^) T-cells are capable to induce GVHD [23]. Thus, it was of importance to elucidate how these T-cell subsets emerge during the early stage of GVHD. To answer this question, the kinetics of donor chimerism as well as the expression of CD44 and CD62 on the splenic T-cells was determined. As shown in Figure 4(a), at the time of T-cell expansion in the GVHD mice (day +5), about 58% of T-cells in the spleen were of donor origin. Phenotype analysis showed that the frequency of effector-memory (CD44^high^CD62^low^) T-cells increased from 17% (day +3) to 52% at day +5, simultaneously the frequency of naïve (CD44^low^CD62^high^) T-cells reduced from 68% (day +3) to 31% at day +5 (Figure 4(d)). Moreover, by progression of GVHD and T-cell infiltration to the tissue (day +21), the frequency of the effector-memory cells remained at same level whereas naïve T-cell population stayed continuously at the lower level (Figure 4(d)). In contrast, in syngeneic recipient mice no increase in effector-memory cell was detected at day +5 or later and naïve T-cells (CD44^low^CD62^high^) were higher than effector-memory (CD44^high^CD62^low^) cells at all evaluated time points (Figure 4(e)).

### 3.7. Histopathologic Evaluation of T-Cells Expansion in the Spleen of GVHD Developing Recipients at Early Time Point

The expansion of CD4+ and CD8+ T-cells in the spleen of allogeneic and syngeneic transplanted mice was examined using immunohistochemistry. As shown in Figure 4(f), CD8+ T-cells expansion is limited to white pulps both in allogeneic and syngeneic transplanted mice three days after BMT. However, 5 days after BMT, the CD8+ cells are spreading all over the spleen (not limited to white pulp) in GVHD developing mice (Figure 4(f)). In sharp contrast, at day +5 after BMT, the number of CD8+ T-cells in the spleen of syngeneic mice dramatically decreases and few existing cells were limited to white pulp (Figure 4(f)). Colonization pattern of CD8+ T-cells return to normal situation in both allogeneic and syngeneic setting 7 days after BMT; however, the population was larger in allogeneic comparing to syngeneic transplanted mice (Figure 4(f)). 

Altogether, these data confirmed results obtained from flow cytometry (Figure 2(d)). The same pattern with less intensity was observed in CD4+ T-cells (data not shown).

### 3.8. Kinetics of Inflammatory Cytokine Production during the Early Stage of GVHD

**Figure 5:**
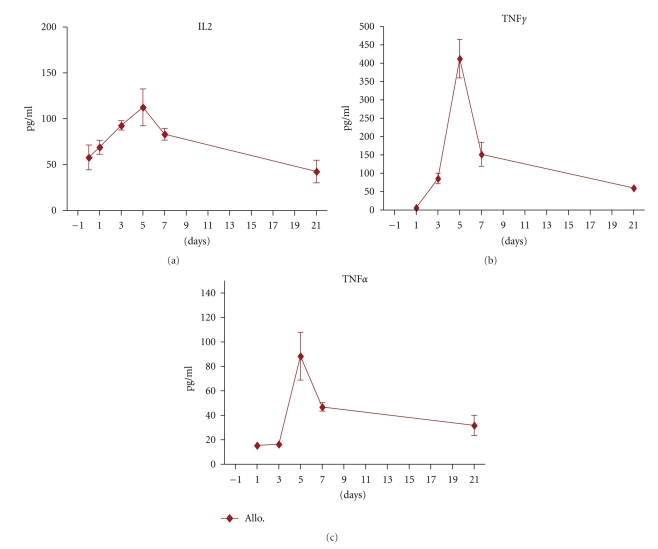
Cytokine levels in the serum of GVHD developing mice. Inflammatory cytokine levels were measured in the serum of allogeneic transplanted BALB/c mice at different time points before and after BMT. (a) IL-2, (b) IFN gamma, and (c) TNF-alfa levels in the serum of GVHD mice. All of the inflammatory cytokines showed maximum level 5 days after BMT in GVHD mice. Serum level of these cytokine was not detectable in syngeneic setting. (*◆*): allogeneic setting Differences were analyzed statistically employing *U*-test, compared between groups (*P < .05).

It is well established that proinflammatory cytokines play a central role in the development of GVHD [20]. The kinetics of IL-2, IFN-*γ*, and TNF-*α* production in the sera of GVHD mice demonstrated (Figure 5(a)) that the serum level of IL-2 increased from 39 ± 13 pg/ml at day −7 (control mice) to 93 ± 8.7 pg/ml (*P* < .05) and 112 ± 20 pg/ml (*P* < .05) at days +3 and +5, respectively, in parallel to DCs and T-cells expansion in the spleen of allogeneic recipients. Syngeneic transplanted mice did not show increment at these time points (data not shown).

IFN-gamma and TNF-alpha are secreted during T-cell proliferation and activation [24–26]. Figures 5(b) and 5(c) represent serum level of IFN-gamma and TNF-alpha in the allogeneic transplanted mice. Both cytokines reach peak serum level at day +5 in GVHD developing mice which are in line with donor T-cell proliferation and activation.

## 4. Discussion

Acute GVHD is a complex inflammatory process in which several factors including conditioning, activation of donor immune cells, and the production of proinflammatory cytokines are suggested to play pivotal roles [1]. Conditioning is an essential prerequisite for HSCT with multiple functions including depletion of hematopoietic stem cells, providing “space” for donor cells, suppression of the host immune system, and most importantly eliminating tumor cells in recipient with malignant disease [27–29]. In the present study, we found that a combination of busulfan and cyclophosphamide (Bu-Cy) as conditioning regimen was able to deplete >95% of both myeloid and lymphoid lineages in the bone marrow. This finding implies that this regimen is myeloablative and thus provides “space” for the donor cells. Bu-Cy regimen induced also a marked decrease (>60%) in the number of splenocytes which suggest that this regimen can also exert a potent immunosuppressive effect. Regarding this issue, we observed Bu-Cy caused a modest (33%) reduction in the number of splenic T-cells and induced a marked decrease in the numbers of B, DC, and NK cells within the range 77%–87% in spleen. This observation clearly implies that these latter immune cells are more sensitive to chemotherapy-based conditioning compared to T-cells. Interestingly murine T-cells were also found to be more resistant to radiation compared to B-cells [30]. Although, the underlying mechanisms for the differential sensitivity of T- and B-cells to chemotherapy- and/or radiation-based conditioning are not well understood, these findings suggest that both regimens share similarities in depletion of immune cells in the recipient.

Several studies have shown that the intensity of the conditioning regimen is positively correlated with the incidence and severity of GVHD which is accompanied by increased damage of the gastrointestinal tract, increased translocation of lipopolysaccaride (LPS) into the circulation and augmented TNF-alfa production [10, 20, 31]. Our finding that at the time of transplantation (day 0), circulatory levels of proinflammatory cytokines, particularly TNF-alfa were low, but markedly increased at the time of T-cell reconstitution in the spleen suggests that in contrast to radiotherapy, chemotherapy-based conditioning plays a minor role in the development of GVHD. Obviously, further studies are required to test this hypothesis.

In the pathogenesis of GVHD, the activation of alloreactive donor T-cells is the hallmark of the disease [2, 31]. The process of activation of alloreactive T-cells is similar to the activation of nonalloreactive antigen specific T-cells [32], that is, they are activated by the antigen presenting cells (APCs), mainly DCs, which express alloantigens [21]. Studies have shown that the presentation of alloantigens to donor T-cells can be performed either by the recipient's [17] or donor's DCs [18, 33, 34]. Using an in vivo tracking model, Beilhack et al. and Panoskaltsis-Mortari et al. have shown the migration pattern of donor cells in the recipient's body. However, both reports did not show the chronobiology and pattern of donor antihost immune cells at the early phase of GVHD [6, 7]. Moreover, in the majority of experimental models the immunobiology of GVHD, the main concern focus on the established picture of disease [5, 17, 18]. To explore the biological role of donor antihost immune cells at the earliest time of GVHD, we studied the phenotypical and expansion pattern of immune cells in the primary and secondary lymphoid organs of GVHD developing mice.

In the present study, the chronological analysis of immune cell reconstitution showed that both host and donor DCs are expanded and activated in the early phase of GVHD. In general, the expansion of host DCs was immediate (day +1) and transient (decelerated by day +3), whereas the expansion of donor DCs was intensive, developed later in the course of GVHD (day +3) which preceded the activation of donor T-cell (day +5) and remained in the expansion phase until the development of clinical manifestations of GVHD. These results suggest that donor DCs might have prominent role (more than expected before) in activation of donor alloreactive T-cells and play a pivotal role in the development of GVHD. Nevertheless more functional studies need to be done. It seems that transient host DCs expansion activate part of donor alloreactive T-cells to recognize peptide-allo-MHC complex, which might lead to the development of a mild GVHD. In contrast, persistent presence of donor DCs continuously activate allorecative T-cells (CD8+) that recognize alloantigens via cross presentation process, which intensifies and perpetuates the development of GVHD. In fact, this possibility is strongly supported by the observation that although GVHD can develop in recipients receiving marrow with major MHC class I-deficiency (beta-2 microglubolin KO mice), but it is strongly potentiated in recipients of bone marrow from wild type donor [18].

During the process of GVHD, activated donor T-cells migrate to target tissue and induce damage via either direct cell contact (cytotoxic T-cells) or cytokine mediated toxicity (T-helper cells) [3, 31]. Despite intensive research about the role of naïve and/or effector-memory T-cells in the induction of tissue damage, only conflicting results are available. For instance, several studies demonstrate that tissue damage in acute GVHD is caused exclusively by activated naïve alloreactive T-cells [35, 36], while others showed that effector-memory alloreactive T-cells are responsible for the tissue destruction [23, 37, 38]. Our study, showed that at the early phase of GVHD (day +3), most of the T-cells have naïve phenotype (CD44^low^CD62^high^) whereas at the late phase (T-cells migration to the tissues, day +5 to day +21), these cells are mainly of effector-memory phenotype (CD44^high^CD62^Low^). These observations imply that upon interaction with host/donor DCs, naïve alloreactive T-cells are firstly activated and thereafter, converted to effector-memory alloreactive T-cells, which are able to migrate to the target tissue and cause damage. This statement is supported by finding that donor CD8+ T-cells recovered on day 42 after allogeneic BMT were mainly of effector-memory phenotype and were able to induce virulent GVHD in secondary recipients [23]. It is valuable to investigate further if the blockade of effector-memory alloreactive T cells can prevent the development of GVHD in our murine model.

A peculiar observation in our study was that five days after the allogeneic BMT, in addition to small and un-granulated lymphocytes, a population of highly granulated lymphocytes emerged in the recipient, which was mainly originated from the donor and consisted of both CD4+ and CD8+ cell populations. Interestingly, the emergence of this population was synchronized with the peak of alloreactive donor cell expansion and with the highest serum levels of inflammatory cytokines. Thus, it is highly possible that these granulated large lymphocytes are responsible for the induction of tissue damage and appearance of clinical manifestations. In fact, our findings that these cells disappeared in the spleen by day +7, in addition to our previous observation that at day +7, alloreactive T-cells migrate to the peripheral tissues [22] support this hypothesis. Indeed, it is important to separate and purify these granulated large lymphocytes and investigate their role in GVHD and more importantly if they can induce GVL. These studies are currently ongoing in our laboratory.

In summary, our results show that GVHD early pathophysiological events following bone marrow transplantation based on busulfan/cyclophosphamide conditioning are similar to that immune response observed in GVHD developed after radiation-based HSCT. However, the rapid kinetics of expansion, proliferation and activation of donor cells that was observed in the present study might be due to the degree of mismatch between donor and recipient. Moreover, the phenotypical changes that occurred during early phase of GVHD (in secondary lymphoid organ) were not detectable among T-cells population after GVHD establishment. Our present model of GVHD based on chemotherapy conditioning regimen is reliable, reproducible, and may give the opportunity to understand mechanisms underlying GVHD in patients conditioned with Bu-Cy compared to that following TBI.

##  Conflict of Interests

The authors have no conflict of interests to declare.

## Figures and Tables

**Table 1:** Effect of conditioning on the different immune cell of bone marrow and spleen. Female BALB/c mice were treated with busulfan (80 mg/kg) followed by cyclophosphamide (200 mg/kg). Bone marrow and spleen cellularity plus immune cell phenotype were evaluated using flowcytometry before and after conditioning. Total and individual immune cells in the bone marrow are more sensitive to the conditioning compare to the spleen cells. ^1^CD3+CD4+, ^2^CD3+CD8+, ^3^CD44^low^CD62^high^, ^4^CD44^high^CD62^low^, ^5^CD19+, ^6^DX5+, ^7^CD11c+, and ^8^CD11b+. (*P < .05)

Time point	Organ	Cellularity	T helper^1^	T cytotoxic^2^	Naïve T cells^3^	Effector memoryT cells^4^	B cell^5^	NK cell^6^	DCs^7^	Myeloid lineage^8^
Before Conditioning (D-7)	BM	39 ± 0.5	0.34 ± 0.02	0.11 ± 0.02	0.05 ± 0.01	0.16 ± 0.01	9 ± 0.6	0.6 ± 0.06	0.44 ± 0.04	15 ± 0.32
After Conditioning (D0)	BM	2 ± 0.2	0.1 ± 0.01	0.08 ± 0.01	0.04 ± 0.01	0.06 ± 0.01	0.13 ± 0.06	0.05 ± 0.01	0.03 ± 0.004	0.2 ± 0.05
*Decrement* * (%)*		95%*	71%*	27%	20%	63%*	99%*	92%*	93%*	99%*

Before Conditioning (D-7)	SP	189 ± 5.6	39 ± 1.7	14 ± 1.5	23 ± 2.1	14 ± 1.1	69 ± 3.5	10.4 ± 1	5 ± 0.3	—
After Conditioning (D0)	SP	70 ± 2.6	26 ± 0.9	12 ± 0.9	19 ± 1.3	7 ± 0.4	16 ± 2.02	1.4 ± 0.2	1 ± 0.1	—
*Decrement* * (%)*		63%*	33%*	14%	17%	50%*	77%*	87%*	80%*	—

*P-value <.01.
